# Description of microbial diversity associated with ticks Hyalomma dromedarii (Acari: Ixodidae) isolated from camels in Hail region (Saudi Arabia) using massive sequencing of 16S rDNA

**DOI:** 10.6026/97320630016602

**Published:** 2020-08-31

**Authors:** Mousa M Alreshidi, Vajid N Veettil, Emira Noumi, Rosa Del Campo, Mejdi Snoussi

**Affiliations:** 1 Department of Biology, College of Science, Ha'il, P.O. 2440, University of Ha'il City 2440, Saudi Arabia; 2 Laboratory of Bioressources: Integrative Biology and Recovery, High Institute of Biotechnology-University of Monastir, Monastir 5000, Tunisia; 3 Servicio de Microbiologia, Instituto Ramon y Cajal de Investigación Sanitaria (IRYCIS), Hospital Universitario Ramon y Cajal, Carretera de Colmenar, Km 9,1, 28034 - Madrid. Spain; 4 Laboratory of Genetics, Biodiversity and Valorization of Bio-resources, Higher Institute of Biotechnology of Monastir, University of Monastir, Avenue Tahar Haddad, 5000 Monastir, Tunisia

**Keywords:** Hyalomma dromedarii, 16S RNA, metagenomic, camels, Hail region

## Abstract

Ticks are blood feeder able to transmit a wide diversity of microbes including pathogens. Therefore, it is of our interest to detect the diversity of microorganisms residing within ticks using massive sequencing of 16S rDNA. In this study, 200 adult ticks were collected from healthy camels in two localities from Hail province (Saudi Arabia). The analysis showed high microbial diversity dominated by the two domains (Archaea and Bacteria) associated with Hyalomma dromedarii from both regions. Proteobacteria (61.3%) and Firmicutes (31.2%) dominated the ticks from the Al Khotha region. While, the microbiome of ticks from the Al Gayed region was dominated by Proteobacteria (81.2%) and Firmicutes (9.2%). Twenty-three families were identified in the DNA-pool from the Al Gayed region, and was dominated by Pseudomonadaceae (45.37%), and Marinobacteraceae (14.39%) families. Francisellaceae (46%), Staphylococcaceae (24.26%) dominated the microbiome of the ticks collected from Al Gayed region. Thus, the genera Pseudomonas, Francisella, Proteus, Marinobacter, Glutamicibacter, Pedobacter, and Staphylococcus are largely distributed in the two identified microbiomes. This study concluded that ticks collected from the studied localities contained a wide range of microbial communities. These data have a great veterinary and medical importance in near future.

## Background

Ticks are great resources for pathogens transmission, including viruses, Rickettsia, bacteria, and protozoa that can produce serious infections in human and animals worldwide [1]. Ticks and mesquites are the most common vectors for pathogens produce human and animal diseases, but ticks are considered the main vector of pathogens harming cattle worldwide [2]. A number of features of ticks enable them excellent vectors of pathogens, include the wide host range and tendency to feed on a number of hosts during life cycle ensure sufficient chance to obtain and pass on microorganisms, toughness and long life make them able to adapt and survive undesirable conditions, high frequency of host-vector contact, the high existing of ticks in both rural and urban areas and long attachment in the host which enable an ample time to transmit pathogenic agents. There is a rising concern in the tick microorganism diversity, as it may induce significant effects of the transmission of pathogenic microbes, and the manipulation of tick microbial community may enhance the pathogens control [3]. Investigations on tick microbes demonstrated their medical and veterinary significance. The spread of tick vectors has led to the increase of tick-borne diseases and microbes associated with ticks [4]. Last decade, the uses of advanced techniques in molecular biology include next-generation sequencing (NGS) enable us to determine the microbial communities within ticks [3,5, 6]. Common pathogens transmitted by ticks are rickettsia, borrelia and Babesia that cause severe infections to both human and animals. Borrelia burgdorferi sensu lato (B. afzelii, B. bavariensis, b gariniiand B. spielmanii. B) causes Lyme diseases (Lyme borreliosis), this bacterium belongs to the spirochaetes and helical- shaped bacteria. It is the main widespread tick-borne disease in US. B. burgdorferiis transmitted to human by blacklegged ticks [7,8]. It was found to be highly existed in the mild climate areas, and it was named after Lyme, a town in Connecticut, USA, where it was first emerged in 1975. Lyme disease is extensively spread, and has been documented in five continents and in more than 70 countries. Symptoms of this disease may include itchiness and redness of the skin, high fever, erythema migrans, neurological disorders and fatigued muscles [6,9,10].

Rickettsia is also a very popular pathogen transmitted by ticks; it is an obligate intracellular Gram-negative bacterium. This bacterium replicates usually in neutrophils and can spread diseases in human and animals [11-13]. The best example of diseases caused by Rickettsiaconorii is rickettsial spotted fever and also known as typhus which highly occurrences in many countries include USA, Asia, and Europe. This disease was first reported in 1936 in Russia. It has been reported that some people with chronic diseases, have underlying diseases [14]. Even though many studies have been conducted to identify microbial communities within ticks worldwide, still a little is known about the microbial diversities inhabiting ticks in Saudi, in particular Ha'il region. Therefore, it is of interest to detect the diversity of microorganisms residing within ticks using massive sequencing of 16S rDNA.

## Methodology

### Study area and tick collection:

The study was conducted in some sites of Hail area. Hail city is located in the middle of the northern region of the Arabian Peninsula, between 25°-29° N and 38°-42° and has an average elevation of 900-1350 m above sea level. It is one of the major cities in the Kingdom of Saudi Arabia and considered as the fifth city in regard to its area. The local geology is dominated by the Arabian shield rock extending to steep wadies and hills. It is characterized by its limestone sand, which exists in the form of sand sheets and sand dunes. The tick samples (200 ticks: 4 to 5 specimen from each animal) were collected from healthy camels from locations of Al Gayed and Al Khotha regions of Hail province. One hundred alive adult ticks were collected from the two studied regions (20 healthy camels from each region). Tick samples were identified by using stereomicroscope and identified based on morphology [15,16]. Pooling was done for engorged ticks from the same camel while ticks were placed individually in 1.5 ml eppendorf tube. Ticks collected were stored at -20°C until DNA extraction.

### DNA extraction and quantification:

The extraction of DNA from tick samples was performed using a commercial DNA extraction kit with the Qiagen DNeasy Tissue kit (Qiagen) following the manufacturer's instructions with some modifications in the initial steps [17]. Briefly, after removal of ethanol,the tick samples were placed in a sterile Petri dish and washed three times in sterile distilled water followed by one absolute ethanol bath, air dried and collected in sterile eppendorf tubes. Followed by washing with phosphate-buffered saline, 300 µl of 0.7M NaOH was added directly to pooled ticks and incubated at 56°C within hot air oven overnight. The ticks were crushed thoroughly using sterile motor and pestles and then the sample tubes were placed in a boiling water bath for 10 min. After cooling, 400 µl of sample solution withOTU disturbing Tick remnants, and placed within a sterile 2mL microcentrifuge tube. The proceeding steps are taken from The QIAamp DNA Mini Kit manufacturer's handbook, following the Tissue Protocol Incubate the samples at 70°C for 10 minutes. The extracted DNA samples were pooled in to two groups based on location. DNA samples were quntified by macrogen. Inc (korea) using picogreen (Invitrogen, cat.#P7589 ) method using Victor 3 fluorometry.

### Sample Preparation:

16S rRNA amplicon sequencing and data analysis:

The 16S RNA hypervariable regions were amplified by using universal primer Bakt-341F and Bakt-805R primers [18]. The genomic libraries for the Illumina sequencing were generated by ligating sequencing adapters and indices to purified PCR products using the Nextera XT Kit (Illumina, San Diego, CA, USA) according to the 16S rRNAmetataxonomics sequencing library preparation protocol (Illumina, San Diego, CA, USA). Followed by equimolar volume of induvidual libraries was pooled and sequenced on an Illumina's Miseq platform with pairedend 300 bp reads by MacrogenInc (Seoul, South Korea). The FLASH version 1.2.11 software was used to assemble MiSeq reads [19]. The taxonomy and alpha diversity were analysed by using QIIME software. The sequencing data is converted into raw data for the analysis. Generation of raw data were done by Illumina sequencer generates raw images utilizing sequencing control software for system control and base calling through an integrated primary analysis software called RTA (Real Time Analysis). The BCL (base calls) binary is converted into FASTQ utilizing illumina package bcl2fastq.

## Results

Table 1 shows the different phylum found in the two pools analyzed. The analyzed pools contained two main domains: Archaea (Euryarchaeota) and Bacteria (eight phylum: Actinobacteria, Bacteroidetes, Cyanobacteria, Deinococcus-Thermus, Firmicutes, Patescibacteria, Proteobacteria, and Verrucomicrobia). Only one domain (Bacteria) is characterizing the pool 1 with only four phylum (Actinobacteria, Bacteroidetes, Firmicutes, and Proteobacteria). Taxonomic profiling showed a high abundance of reads assigned to the phylum Proteobacteria in both pools analyzed. Firmicutes phylum is abundant in the first pool with more than 29540 OTU ID identified as compared to the second pool (3231 OTU ID). All these data are summarized in Table 1.

Twenty-three families (Table 2) were identified in DNA-pool 1 represented mainly by Pseudomonadaceae (30828 OTU ID, 45.37%), Marinobacteraceae (9777 OTU ID, 14.39%), Enterobacteriaceae (7954 OTU ID, 11.71%), Micrococcaceae (4246 OTU ID, 6.25 %), Sphingobacteriaceae (3257 OTU ID, 4.79%), and Staphylococcaceae (3108 OTU ID, 4.57%).

**Figure 1:**
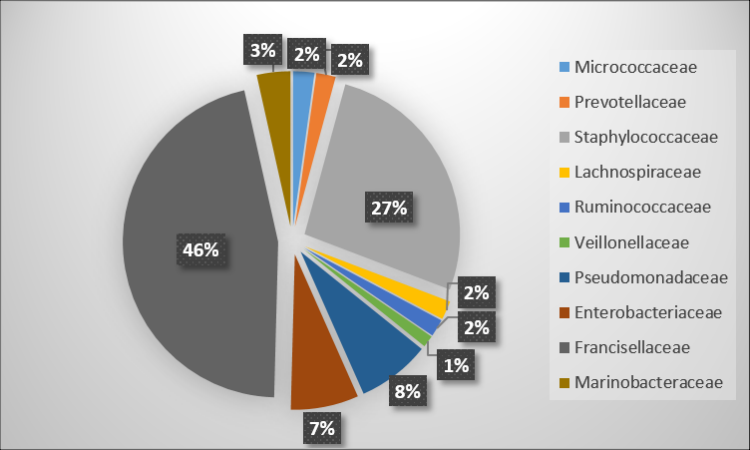
Distribution of the main families in DNA pool 1. The microbiome of the second DNA-pool contains also several bacterial families with human health interest (Figure 2) including Christensenellaceae (1.01 %), Moraxellaceae (0.90 %), Rikenellaceae (0.87 %), Sphingobacteriaceae (0.60 %), and Xanthomonadaceae (0.51 %)

In the second DNA pool from Al Khotha region, 49 families were identified represented mainly by Francisellaceae (46%), Staphylococcaceae (24.26%), Pseudomonadaceae (6.92%), Enterobacteriaceae (6.45%), and Marinobacteraceae (3.20%). The results are summarized in Figure 1.

Results showed the identification of 37 genera in the microbiome of DNA-poo1 from Al Gayed region and 84 genera in DNA-pool 2 from Al Khotha. The first DNA pool from Al Gayed region is dominated by the following genera: Pseudomonas (45.37%), Marinobacter (14.39 %), Proteus (7.98 %), Glutamicibacter (6.15 %), Pedobacter (4.79 %), and Staphylococcus (3.85 %), Enterobacteriaceae unidentified genera (3.31 %), Stenotrophomonas(2.61 %),Francisella(2.52 %), Psychrobacter(1.59 %), and Brevibacterium(1.46 %).While the pool 2 microbiome contains mainly: Francisella (42.09 %), Staphylococcus (22.83 %), Pseudomonas (6.92 %), Proteus (5.99 %), and Marinobacter (3.20 %) genera. These results are summarized in Table 3.

**Figure 3:**
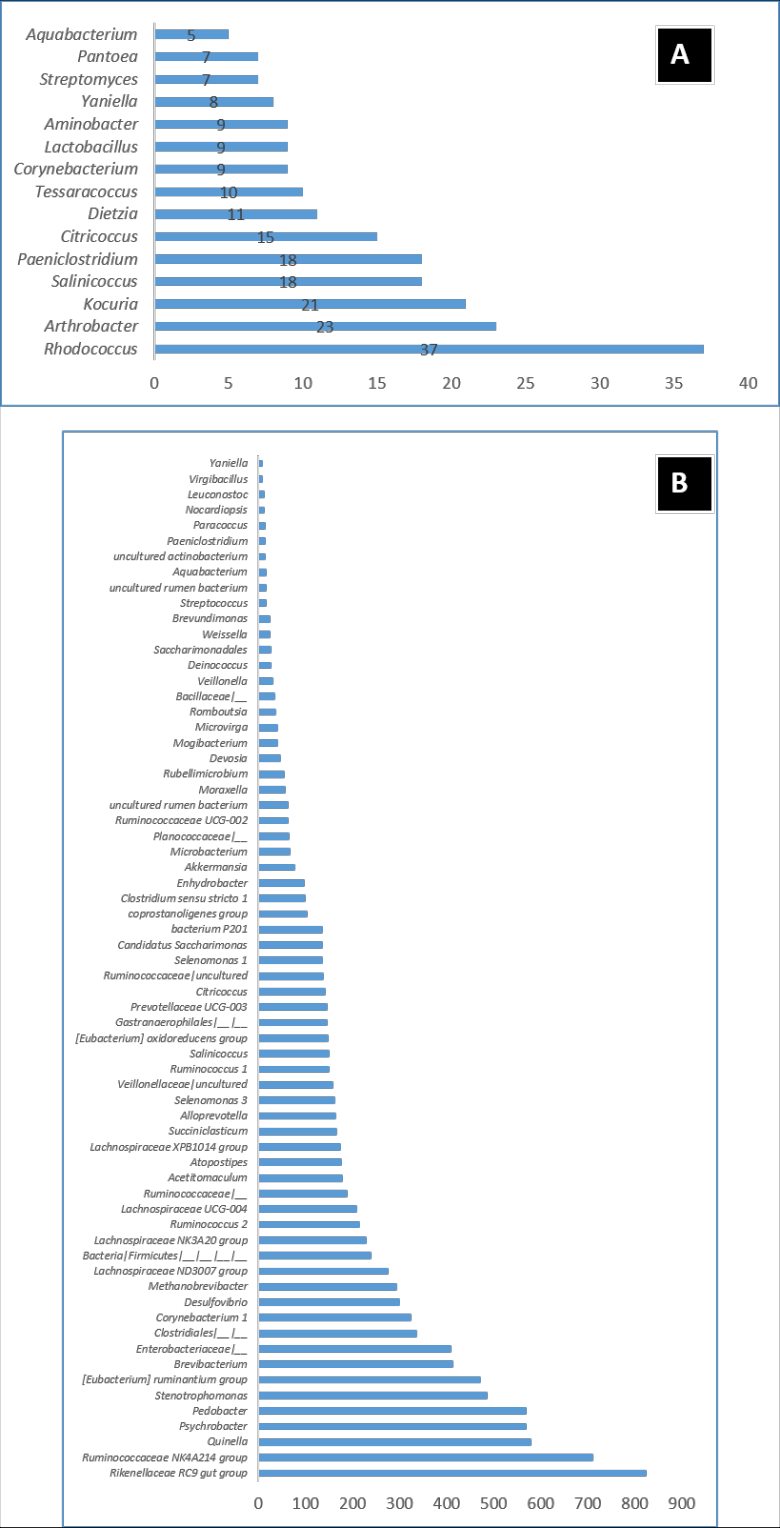
Distribution of the genera identified in the microbiome of DNA-pool 1 (Al Gayed region) according the number of OTU identified.

Different other genera have been identified less distributed in the two microbiomes with percentage varying from (0.05 to 0.01 %) in the DNA-pool 1 including Rhodococcus, ArthrobacterKocuria, Salinicoccus, Paeniclostridium, Citricoccus, Dietzia, Tessaracoccus, Corynebacterium, Lactobacillus, Aminobacter, Yaniella, Streptomyces, Pantoea, Aquabacterium (Figure 3A). In the DNA-pool 2, more than 66 genera were identified with a percentage of distribution ranging from 0.87% to 0.01%. These genera include among others: Rikenellaceae RC9 gut group, Ruminococcaceae NK4A214 group, Quinella, Psychrobacter, Pedobacter, Stenotrophomonas, [Eubacterium] ruminantium group, and Brevibacterium (Figure 3B).

## Discussion

It is well known that Hyalomma dromedarii ticks are a vector of pathogenic and endobiotic bacteria, fungi, virus (especially, Middle East respiratory syndrome coronavirus; MERS-CoV), and protozoan in different regions in Saudi Arabia [20-24]. These hard ticks are frequently associated with camels (Camelus dromedaries) worldwide, and especially in temperate regions (North Africa, Middle East, Arabian Gulf and Asia), which affect their behavior, health, productivity and performance [22,25-27]. In Egypt, there is not enough available data about Q fever in camels linked with their tick vectors. The study of Abdullah et al. [28] focused on the detection of the Q fever Coxiellaburnetii in camels and ixodid ticks using PCR and sequencing of the targeting IS30A spacer. The same team evaluated 16S ribosomal RNA (16S rRNA) and cytochrome oxidase subunit-1 (CO1) genes using PCR and sequencing tools as molecular methods and sodium dodecyl sulfate-polyacrylamide gel electrophoresis (SDS-PAGE) and western blot as immunological methods for characterization of the two tick species H. dromedarii and H. excavatum. The ixodid ticks are vectors for bacterial and viral pathogens and many protozoans. For this, the first step of the study of Abdullah et al. [28] was to investigate the presence of Q fever in camels and related ticks using PCR and sequencing analyses [28]. Furthermore, PCR and sequencing were used to start the second step related to the examination of the two genes 16S rRNA and CO1 for molecular characterization of the camel tick species (H. dromedarii and H. excavatum). The selection of 16S rRNA and CO1 genes was based on the studies of Chitimia et al. [29] and Lv et al. [30] demonstrating that DNA markers are more reliable in discriminating species of ticks [29].

Most previous studies of H. dromedarii in Saudi Arabia have focused on viral and protozoan pathogens but not on bacterial agents [31,32]. Moreover, most of the studies used species-specific PCR-based assay for the screen of H. dromedarii-borne bacteria. Currently, the 16S rRNAmetataxonomics analyses circumvent the limitation of previous methods, facilitating detection of more bacterial communities in ticks. The tick microbiome of the genus Hyalomma has only been characterized in the species H. rufipes, H. annotilucm, H. isaaci, H. scupense, H. aegyptium, H. marginatum and H. excavatum by 16S rRNAmetataxonomic approach [33-35]. In Saudi Arabia, few studies have used the metagenomic approach to describe the microbial diversity of ticks (H. dromadarii) associated with camels [36]. This approach facilitates the identification of more microorganisms as compared to the PCR-based techniques targeting specific known pathogens. In fact, using amplification of V3-V4 region of 16S rRNA from ticks, Elbir and colleagues [36] showed that the microbiome prepared from H. dromedarii ticks associated with camels in Hofuf city was dominated by Proteobacteria phylum with more than 98% of the identified microorganisms followed by Firmicutes (1.38%), Actinobacteria (0.36%), Bacteroidetes (0.17%). Our results showed similar results with higher bacteriome diversity in the two pools tested with dominance of Proteobacteria (61.29-81.22%), Firmicutes (4.75-31.24%), Bacteroidetes (3.54-4.79%), and Actinobacteria (3.18-9.22%). The same team reported similar results with phyla Cyanobacteria, Verrucomicrobia, detected in low percentages in the present study (0-0.15% and 0-0.08%, respectively). Additionally, our results showed the identification of 96 genera in two pools tested. In fact, Elbir et al.[36] reported the identification of 114 genera with more than 217 species highlighting the microbial diversity of population infecting camel ticks. Francisellaceae, Staphylococcaceae, Pseudomonadaceae, and Enterobacteriaceae were the dominant bacterial families identified in the tested H. dromaderii from Hail city. Additionally, Elbir et al.[36] have reported similar results from Hofuf city (Eastern region). These authors reported that the endosymbiotic bacteria belonging to Francisella genus (94.37%) dominated H. dromedarii ticks.

In the present study, we didn’t detected Borrelia and Rickettsia pathogens in the two tested H. dromedarii DNA-pools. Similar results have been reported by Elbir et al. [36] from Hufouf region. Many bacterial genus can be detected in the tick specie H. dromedarii. The variability of the bacterial types in tick genera results from differences in their ecological location, the host genotype and the health status. The most dominant bacterial genera in the tick species were Clostridium, Corynebacterium, Staphylococcus and Ralstonia [34, 37]. Rickettsia, Francisella and Coxiella were the most important tick bacterial species that were identified associated with tick species infesting livestock in Pakistan [38]. The presence of Rickettsia was further explored by PCR amplification of the SFGR-specific ompA gene [39]. Elbir et al.[36] conducted analysis for bacterial community in whole H. dromedarii ticks in Saudi Arabia via sequencing of the V3-V4 segment of 16S rRNA gene using Illumina MiSeq sequencer. These authors demonstrated that the most dominant bacterial types were Proteobacteria (98.12%) represented by the genus Francisellawith average abundance of 94.37% followed by Proteus (2.97%) and Acinetobacter (0.46%) and Pseudomonas (0.14%). The second bacterial type was Firmicutes (1.34%) represented by the genus Staphylococcus (0.51%), Salinicoccus (0.21%), Enterococcus (0.12%) and Solibacillus (0.1%). Actinobacteria (0.33%) was the third bacterial type represented essentially by Corynebacterium (0.25%) and the last one was the Bacteroidetes (0.16%). This study concluded that ticks associated with camels collected from Hail region (Saudi Arabia) had a wide diversity of microorganisms belonging mainly to the domains Archaea and Bacteria. Using the metagenomic approach, more than nine phylum, 53 families, and 94 genera were identified in the two DNA-pools studied. The obtained results highlighted the medical importance of ticks (H. dromedarii) as reservoir of pathogenic, opportunistic and symbiotic microorganisms. A program of veterinary surveillance must be programmed in order to identify known pathogenic microorganisms associated with H. dromedarii in Hail region.

## Funding:

Scientific Research Deanship at University of Ha'il-Saudi Arabia has funded this research through project number (Grant No. BA-1510)

## Declaration on Publication Ethics:

The authors state that they adhere with COPE guidelines on publishing ethics as described elsewhere at https://publicationethics.org/. The authors also undertake that they are not associated with any other third party (governmental or non-governmental agencies) linking with any form of unethical issues connecting to this publication. The authors also declare that they are not withholding any information that is misleading to the publisher in regard to this article.

The authors are responsible for the content of this article. The Editorial and the publisher has taken reasonable steps to check the content of the article with reference to publishing ethics with adequate peer reviews deposited at PUBLONS.

## Figures and Tables

**Table 1:** Distribution of domains and phylum in the two pools of ticks DNA analyzed

#OTU ID	Pool 1 (Al Gayed Region)		Pool 2 (Al Khotha Region)	
	Number of OTU	%	Number of OTU	%
Archaea|Euryarchaeota	0	0	293	0.31
Bacteria|Actinobacteria	6269	9.226	3007	3.181
Bacteria|Bacteroidetes	3257	4.793	3346	3.539
Bacteria|Cyanobacteria	0	0	146	0.154
Bacteria|Deinococcus-Thermus	0	0	26	0.028
Bacteria|Firmicutes	3231	4.755	29540	31.246
Bacteria|Patescibacteria	0	0	161	0.17
Bacteria|Proteobacteria	55192	81.226	57946	61.292
Bacteria|Verrucomicrobia	0	0	76	0.08
Total OTU identified	67949	-	94541	-

**Table 2:** Identified families in the DNA-pool 1 from Al Gayed region

Families identified	OUT number	%
Pseudomonadaceae	30828	45.37
Marinobacteraceae	9777	14.39
Enterobacteriaceae	7954	11.71
Micrococcaceae	4246	6.25
Sphingobacteriaceae	3257	4.79
Staphylococcaceae	3108	4.57
Xanthomonadaceae	1776	2.61
Francisellaceae	1710	2.52
Moraxellaceae	1539	2.26
Burkholderiaceae	1045	1.54
Brevibacteriaceae	994	1.46
Halomonadaceae	554	0.82
Corynebacteriaceae	410	0.6
Microbacteriaceae	362	0.53
Dermabacteraceae	192	0.28
Bacillaceae	96	0.14
Nocardiaceae	37	0.05
Peptostreptococcaceae	18	0.03
Dietziaceae	11	0.02
Propionibacteriaceae	10	0.01
Lactobacillaceae	9	0.01
Rhizobiaceae	9	0.01
Streptomycetaceae	7	0.01

**Table 3:** Distribution of the main genera in the microbiome of the two tested DNA-pools

Genera Identified	Pool 1		Pool 2	
	Number of OUT	%	Number of OUT	%
Pseudomonas	30828	45.37	6543	6.92
Marinobacter	9777	14.39	3024	3.2
Proteus	5420	7.98	5663	5.99
Glutamicibacter	4179	6.15	1760	1.86
Pedobacter	3257	4.79	568	0.6
Staphylococcus	2617	3.85	21587	22.83
Enterobacteriaceae genera	2249	3.31	409	0.43
Stenotrophomonas	1776	2.61	486	0.51
Francisella	1710	2.52	39792	42.09
Psychrobacter	1081	1.59	569	0.6
Brevibacterium	994	1.46	413	0.44
Halomonas	554	0.82	291	0.31
Pusillimonas	553	0.81	222	0.23
Jeotgalicoccus	473	0.7	1195	1.26
Acinetobacter	458	0.67	130	0.14
Corynebacterium 1	401	0.59	0	0
Leucobacter	362	0.53	99	0.1
Burkholderiaceaegenera	334	0.49	92	0.1
Enterobacter	278	0.41	92	0.1
Brachybacterium	192	0.28	173	0.18
Parapusillimonas	153	0.23	57	0.06
Bacillus	96	0.14	98	0.1
Prevotella 1	0	0	1436	1.52
ChristensenellaceaeR-7 Group	0	0	951	1.01

**Figure 2:**
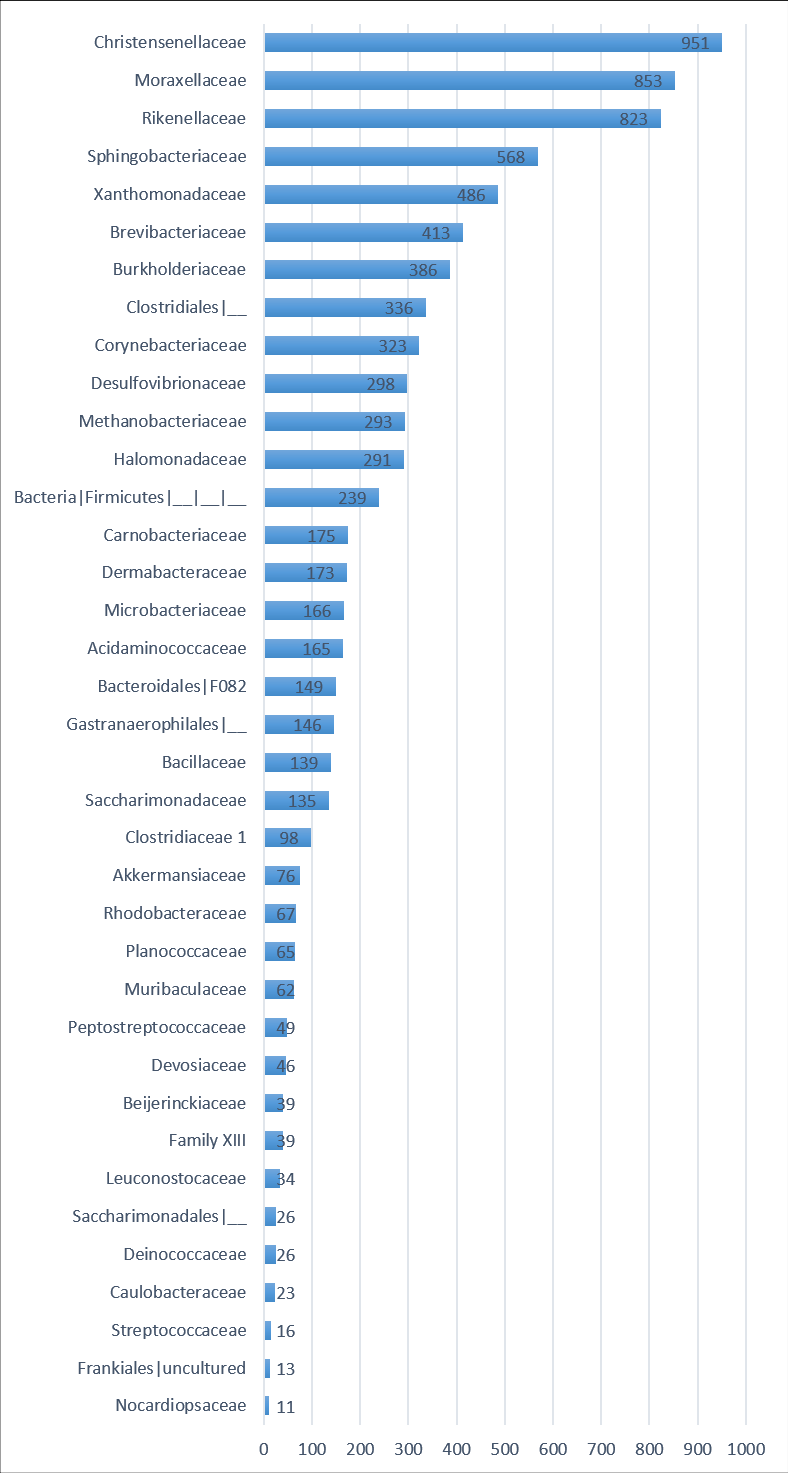
Distribution of the families in DNA pool 2 from AL Khotha region.
